# Effect of potassium fertilization on storage root number, yield, and appearance quality of sweet potato (*Ipomoea batatas* L.)

**DOI:** 10.3389/fpls.2023.1298739

**Published:** 2024-02-22

**Authors:** Ben-kui Liu, Bing-jie Xv, Cheng-cheng Si, Wen-qing Shi, Guo-zheng Ding, Li-xue Tang, Ming Xv, Chun-yv Shi, Hong-jvan Liu

**Affiliations:** ^1^ College of Agronomy, Shandong Agricultural University, Tai’an, Shandong, China; ^2^ School of Breeding and Multiplication (Sanya Institute of Breeding and Multiplication), Hainan University, Sanya, China; ^3^ Shandong Agricultural Technology Extension Center, Jinan, Shandong, China; ^4^ Shandong Academy of Agricultural Sciences, Jinan, Shandong, China

**Keywords:** sweet potato, potassium application, lignin metabolism, storage root number, storage root uniformity

## Abstract

Increasing storage root number is a pivotal approach to enhance both storage root (SR) yield and appearance quality of sweet potato. Here, 2-year field experiments were conducted to investigate the effect of 0 (K0), 120 (K1), 240 (K2), and 360 (K3) kg ha^−1^ potassium fertilizer (K_2_O) on lignin metabolism, root growth, storage root yield, and uniformity. The results demonstrated that potassium (K) application led to a decrease in the activities of key enzymes involved in lignin biosynthesis, including phenylalanine deaminase (PAL), 4-coumarate coenzyme A ligase (4-CL), cinnamic acid dehydrogenase (CAD), polyphenol oxidase (PPO), and peroxidase (POD). This resulted in a significant reduction in lignin and G-type lignin contents in potential SRs compared to K0 treatment within 10–30 days after planting (DAP). BJ553 exhibited a significant decrease in PAL activity, as well as lignin and G-type contents at 10 DAP, whereas YS25 showed delayed effects until 20 DAP. However, the number and distribution of secondary xylem conduits as well as the mid-column diameter area in roots were increased in K2 treatment. Interestingly, K2 treatment exhibited significantly larger potential SR diameter than other treatments at 15, 20, and 25 DAP. At harvest, K2 treatment increased the SR number, the single SR weight, and overall yield greatly compared with K0 treatment, with an average increase of 19.12%, 16.54%, and 16.92% respectively. The increase of SR number in BJ553 was higher than that of YS25. Furthermore, K2 treatment exhibited the lowest coefficient of variation for both SR length and diameter, indicating a higher yield of middle-sized SRs. In general, appropriate potassium application could effectively suppress lignin biosynthesis, leading to a reduction in the degree of pericycle lignification in potential SRs. This promotes an increase in the number of storage roots and ultimately enhances both yield and appearance quality of sweet potato. The effect of potassium fertilizer on lignin metabolism in BJ553 roots was earlier and resulted in a greater increase in the SR number compared to YS25.

## 1 Introduction

Sweet potato [*Ipomoea batatas* (L.) Lam] is one of the world’s seven major food crops; China accounts for 22.94% of world area harvested but 30.12% of world production (FAOSTAT, 2022). The high nutritional content of fresh sweet potatoes, including carbohydrates, vitamins, dietary fiber, and essential minerals, contributes to their immense popularity among consumers. The substantial market demand has necessitated heightened quality standards for storage roots (Low et al., 2015; Singh et al., 2021; Soto-Caro et al., 2022). The appearance quality of storage roots is the primary factor that directly influences both the sales price and consumer demand, among factors including nutrition and taste quality (Soto-Caro et al., 2022). Appearance quality includes skin color, size, shape, and uniformity of SRs (Anderson et al., 2021; Lado et al., 2021), and the size and uniformity greatly influence profitability, especially when sold by grade. The lack of emphasis on appearance quality, however, has resulted in inconsistent sizes and a significant number of substandard SRs, thereby compromising production efficiency and impeding the further advancement of fresh sweet potatoes.

Increasing SR number is an effective way to improve uniformity of SR, as demonstrated by cultivation measures including plant density, water management, and fertilizer application (Yan et al., 2017). The differentiation direction of adventitious roots is determined by the procambium activity and lignification degree of pericycle (Tanaka et al., 2005; Tanaka, 2016). The formation of SRs is facilitated by high procambium activity and low lignification degree. The activity of procambium is primarily associated with cellular proliferation and differentiation, necessitating an adequate supply of energy (Villordon et al., 2009). Fertilization can increase the content of energy substrates such as hexose in adventitious roots, thereby increasing the number of SRs per plant (Si et al., 2018; Du et al., 2020; Si et al., 2022). Lignin metabolism is closely related to lignification degree of pericycle (Ohashi-Ito et al., 2010), while the deposition of lignin, cellulose, and hemicellulose in the secondary cell wall has been implicated in regulating vascular development; however, their effect on SR formation remains controversial. The previous study showed that SR formation is associated with the downregulation of genes involved in lignin biosynthesis and a decrease in lignin accumulation (Firon et al., 2013). However, the present study showed that xylem development is accompanied by lignin accumulation of the proximal part of the root during SR formation (Singh et al., 2021). Dipping seedlings with gibberellin significantly upregulated key genes and potential upstream regulators involved in lignin biosynthesis, leading to increased lignin levels and reduced the number of SRs (Singh et al., 2019). Conversely, paclobutrazol increased SR numbers and yield by modulating phytohormones and lignin biosynthesis in sweet potato (Si et al., 2023). Potassium application under cadmium stress conditions enhances lignin accumulation in the root tip of sweet potato (Yin et al., 2022). However, the regulatory mechanism of potassium on lignin metabolism during SR formation of roots remains unclear. Therefore, we conducted a 2-year field experiment with four levels of potassium fertilizer to evaluate the effects of K fertilizer on the morphological characteristics, tissue structure, and lignin metabolism of the adventitious roots during SR formation in sweet potato. Additionally, we investigated the interrelationships among lignin metabolism, adventitious root differentiation, and the number of SRs per plant. Furthermore, we examined how the quantity of SRs affects their appearance quality.

## 2 Materials and methods

### 2.1 Experimental site

Field experiments were conducted in 2017–2018 by using two sweet potato cultivars Beijing 553 (BJ553) and Yanshu 25 (YS25) at the Agronomy Experimental Station of Shandong Agricultural University, Tai’an City, Shandong Province (36°09′N, 117°09′E). Both YS25 and BJ553 are used as the main edible sweet potato; YS25 generally had three to four storage roots, which was higher than that of Beijing 553. Climate data for the two growing seasons were provided by the Tai’an Meteorological Bureau of Shandong and automatic weather station in the Agronomy Experimental Station (**Table 1**). The soil texture of the experimental field was sandy loam, and the organic matter content of the 0–20 cm soil layer was 1.08%, alkaline N 79.47 mg kg^−1^, available phosphorus 27.01 mg kg^−1^, and available potassium 78.21 mg kg^−1^ in 2017; the organic matter content of the 0–20 cm soil layer was 1.03%, alkaline N 76.42 mg kg^−1^, available phosphorus 26.36 mg kg^−1^, and available potassium 86.02 mg kg^−1^ in 2018.

**Table 1:** Climate data for the two growing seasons of sweet potato.

Month	2017		2018	
	Average Temperature (°C)	Precipitation (mm)	Average Temperature (°C)	Precipitation (mm)
5	23.79	24.1	24.30	58
6	25.45	57.2	27.58	77.5
7	28.69	236.8	30.00	39
8	28.00	75.2	25.16	283.5
9	23.25	31.4	21.57	75.7
10	14.48	27.3	13.90	6

### 2.2 Experimental design

The field experiments were conducted using a split plot design with three replications. Each subplot of BJ553 and YS25 cultivars consisted of four different concentrations of potassium fertilizer (K_2_O): 0, 120, 240, and 360 kg ha^−1^ represented as K0, K1, K2, and K3, respectively. Potassium sulfate (51% K_2_O) and urea (46% N) were used as base fertilizers. Nitrogen (N) fertilizer was applied at 90 kg ha^−1^ for all treatments. The fertilizers were provided by Sinofert Holdings Limited (Beijing, China). Sweet potatoes were planted at 0.75 m row spacing and 0.25 m plant spacing in row, with a plot area measuring 15 m^2^. Sweet potatoes were planted on 12 May 2017 and 13 May 2018 respectively, and harvested on 2 October 2017 and 17 October 2018, respectively.

### 2.3 Sampling

Approximately six to eight plants were selected for each treatment at 10, 15, 20, 25, and 30 days after planting. The roots were thoroughly excavated, rinsed with cold distilled water, and the potential storage roots were promptly and accurately selected at 0°C. Subsequently, they were hermetically sealed, flash-frozen in liquid nitrogen, and stored at −80°C for subsequent determination of lignin content and its associated enzyme activities.

### 2.4 Measurements

#### 2.4.1 Root traits

Approximately six to eight plants were selected for each treatment at 15, 20, and 25 DAP. Subsequently, all roots was dug out carefully, then the number of potential storage roots was counted manually, and diameters were measured with vernier calipers, after that the average was calculated.

#### 2.4.2 Anatomical structure

Roots were sampled from six to eight plants from each treatment. Select the thickest root from each plant and make a 0.5-cm incision at the top 2 cm of the root. Five roots were chosen to make the sections. The sections were made using the paraffin sectioning method, stained with safranin-fast green and phloroglucin reagent according to the method of Belehu et al. (2004). Phloroglucinol can selectively stain the lignin of cells. The root and stele diameters, as well as the number of secondary xylems, were measured in three sections for each dyeing method and treatment using NIS-Elements software. The collected data were then processed in Excel 2016.

#### 2.4.3 Lignin content and related enzyme activities

The total lignin content was determined according to the method of Lin et al. (2012). The content of lignin monomers (S, G, and H subunits) was determined following Zheng et al. (2017). Phenylalanine ammonialyase (PAL), 4-coumaric acid: CoA ligase (4-CL), cinnamyl alcohol dehydrogenase (CAD), and peroxidase (POD) activities were determined by the method of Kamran et al. (2018). Polyphenol oxidase (PPO) activity was assayed according to the method described by Aquino-Bolanos and Mercado-Silva (2004).

#### 2.4.4 Storage root number, yield, and appearance quality

All SRs were dug out to count the SR number (root diameter more than 2.0 cm) of each plant and measure SR weight in each plot at harvest time. SRs of 20 plants for each plot were selected for commercial SR grading (>100 g is commercial storage root, >500 g is large storage root, 150–500 g is medium storage root, and <150 g is small storage root) and SR length and diameter measurements. Then, the uniformity of SRs and the rate of commercial SRs were calculated.

### 2.5 Statistical analysis

The data, after removing outliers, were analyzed using Statistix 10 (Statistix Software Inc., USA, www.statistix.com). The yield data were analyzed by two-way ANOVA, and the other data were analyzed by one-way ANOVA based on new Duncan’s at a 0.05 or 0.01 probability level. The figures were designed by Origin Pro 2021 (OriginLab, Inc. USA, https://www.originlab.com/).

## 3 Results and analysis

### 3.1 Effects of potassium application on yield and uniformity of sweet potato storage roots at harvest time

#### 3.1.1 Storage roots yield

Compared with K0 treatment, K2 treatment significantly increased SR number per plant, the mean fresh weight of single SR, and yield. The average number of SR per plant was higher in BJ553 compared to YS25, while the average increase of yield and mean single SR weight over 2 years was lower. However, the increases of yield and its components would decrease if the potassium application were insufficient or excessive (**Table 2**). The ANOVA analysis showed that the impact of potassium fertilizer is more significant than variety and their interaction.

**Table 2:** Sweet potato storage root yield and its components.

Years	Cultivars	Treatment	Storage root no. per plant	Mean fresh weight of single storage root (g)	Storage roots yield (t ha^−1^)
2017	YS25	K0	2.52 d	286.56 e	39.56 c
		K1	2.75 c	304.47 d	41.23 c
		K2	3.16 a	341.81 a	46.41 a
		K3	2.93 b	334.22 ab	44.46 b
	BJ553	K0	1.51 g	292.96 de	22.63 e
		K1	1.69 f	311.82 de	23.89 e
		K2	2.01 e	328.95 bc	26.81 d
		K3	1.94 e	319.61 c	26.50 d
ANOVA analysis					
Varieties			1567.44**	67.72**	37.13**
Potassium			87.58**	8.37**	1843.31**
Varieties × Potassium			1.76	3.37*	1.98
2018	YS25	K0	4.59 c	137.02 d	34.07 e
		K1	4.74 b	152.22 cd	37.14 bc
		K2	4.94 a	165.89 c	39.09 a
		K3	4.80 b	163.20 c	39.84 a
	BJ553	K0	3.00 f	212.06 b	34.85 de
		K1	3.12 e	225.75 ab	36.22 cd
		K2	3.31 d	240.72 a	40.06 a
		K3	3.20 e	233.91 ab	38.47 ab
ANOVA analysis					
Varieties			4963.48**	162.02**	0.10
Potassium			36.03**	4.83**	31.75**
Varieties × Potassium			0.17	0.03	2.09

K0, K1, K2, and K3 indicated that K fertilizer rates were 0, 120, 240, and 360 kg ha^−1^, respectively. For each cultivar, values followed by the different letters within the same column are significantly different among potassium treatments at the 0.05 probability level. * p < 0.05; ** p < 0.01.

#### 3.1.2 Commercial storage root yield

Potassium application rate significantly affected the commercial SR yield of sweet potato in our experiment. Compared with K0 treatment, potassium application increased the commercial SR yield, improved the quality ratio of commercial SRs, and increased the rate of small and medium-sized SRs, while decreasing the rate of large ones. Among all treatments, K2 treatment resulted in the highest weight for medium-sized storage roots but the lowest weight for large ones. Both varieties showed consistent trends in response to potassium application, but BJ553 exhibited greater variation than YS25 (**Table 3**).

**Table 3:** Commercial storage root weight ratio and their weight ratio of large, medium, and small storage roots (2018).

Cultivar	Treatment	CSRY (t ha^−1^)	CSRWR (%)	LSRW (t ha^−1^)	LSRWR (%)	MSRW (t ha^−1^)	MSRWR (%)	SSRW (t ha^−1^)	SSRWR (%)
YS25	K0	32.28 c	94.75	12.18 a	35.75	18.86 c	55.36	3.03 c	8.88
	K1	35.25 b	94.92	9.32 b	25.09	21.66 b	58.33	6.16 a	16.58
	K2	37.18 a	95.24	6.09 c	15.61	28.75 a	73.66	4.19 b	10.73
	K3	38.09 a	95.49	11.22 a	28.13	22.24 b	55.76	6.43 a	16.11
BJ553	K0	34.30 b	95.98	21.55 a	61.06	12.48 c	34.93	1.43 c	4.01
	K1	35.20 b	97.17	21.69 a	59.64	12.67 c	34.97	1.95 b	5.39
	K2	39.48 a	98.55	14.58 b	36.08	22.58 a	56.36	3.02 a	7.55
	K3	35.55 b	95.59	14.24 b	39.03	20.29 b	55.13	2.15 b	5.84

K0, K1, K2, and K3 indicated that K fertilizer rates were 0, 120, 240, and 360 kg ha^−1^, respectively. >100 g is commercial storage root, > 500 g is large storage root, 150–500 g is medium storage root, and <150 g is small storage root. CSRY, commercial storage root yield; CSRWR, commercial storage root weight ratio; LSRW, large storage root weight; MSRW, medium storage root weight; SSRW, small storage root weight; LSRWR, large storage root weight ratio; MSRWR, medium storage root weight ratio; SSRWR, small storage root weight ratio. For each cultivar, values followed by different letters within the same column are significantly different among potassium treatments at the 0.05 probability level.

#### 3.1.3 Uniformity of storage roots

The smaller coefficient of variation indicates a lower level of discreteness and a higher degree of uniformity in the data (Abdi, 2010; Lorenzo et al., 2015). Data in **Table 4** showed that potassium application significantly increased the weight and length of SRs, but decreased the coefficient of variation of SR length and diameter compared to K0 treatment. The variations in the coefficient of variation for the length and diameter of SR in YS25 were significantly greater compared to those observed in BJ553. The significant changes all appeared in K2 treatment (**Table 4**).

**Table 4:** Uniformity of storage roots at harvest time (2018).

Cultivar	Treatment	Fresh weight of storage root (g)	*CV* (%)	Storage root length (cm)	*CV* (%)	Storage root diameter (mm)	*CV* (%)
YS25	K0	137.02 d	13.86	22.33 c	26.73	44.39 c	43.09
	K1	152.22 c	11.78	24.00 ab	19.12	50.23 b	28.16
	K2	160.84 a	8.72	24.50 a	14.68	53.57 a	18.85
	K3	156.02 b	9.24	23.50 b	26.09	49.92 b	25.16
BJ553	K0	202.74 d	12.05	19.46 c	28.33	70.28 a	28.83
	K1	225.75 c	6.03	22.46 ab	22.09	65.45 b	26.58
	K2	239.59 a	5.16	23.79 a	20.56	55.40 c	25.61
	K3	214.63 c	7.76	21.39 b	23.84	66.21 b	27.78

K0, K1, K2, and K3 indicated that K fertilizer rates were 0, 120, 240, and 360 kg ha^−1^, respectively. For each cultivar, values followed by different letters within the same column are significantly different among potassium treatments at the 0.05 probability level.

### 3.2 Effect of potassium application on potential storage roots traits during storage root formation

The number and diameter of potential storage roots in both cultivars responded similarly to potassium during storage root formation in both experimental years (**Table 5**). The number of potential SRs significantly increased in the potassium treatment compared to K0, with the greatest increase observed in K2 at 15, 20, and 25 DAP. K2 treatment also increased diameter of potential SRs. The response of BJ553 to potassium fertilizer was more evident in root number, while the response of YS25 was more pronounced in root diameter.

**Table 5:** Number and diameter of potential storage roots during storage root initiation.

Year	Cultivar	Treatment	15 days		20 days		25 days	
			No.	Diameter (cm)	No.	Diameter (cm)	No.	Diameter (cm)
2017	YS25	K0	33.40 c	1.03 b	35.60 b	1.28 a	20.00 c	2.00 c
		K1	37.40 a	1.08 a	43.20 a	1.31 a	22.60 ab	2.33 b
		K2	39.60 a	1.14 a	46.40 a	1.35 a	25.40 b	2.74 a
		K3	33.20 b	1.12 a	43.60 a	1.35 a	20.80 bc	2.27 b
	BJ553	K0	60.80 c	0.90 a	39.60 c	1.05 c	45.60 b	1.30 b
		K1	72.60 b	0.93 a	51.40 b	1.08 c	51.00 a	1.35 ab
		K2	84.40 a	0.96 a	59.00 a	1.12 b	53.20 a	1.36 ab
		K3	60.60 c	0.93 a	48.00 b	1.28 a	49.20 ab	1.42 a
2018	YS25	K0	40.75 b	0.89 c	29.00 a	1.05 c	49.60 b	1.62 a
		K1	49.80 a	1.04 b	33.20 a	1.06 c	52.00 ab	1.66 a
		K2	52.80 a	1.17 a	33.40 a	1.21 a	53.80 a	1.64 a
		K3	49.20 a	1.11 ab	33.00 a	1.14 b	52.60 ab	1.64 a
	BJ553	K0	52.20 b	0.71 b	34.20 b	0.77 b	48.60 b	0.93 c
		K1	53.00 b	0.68 b	39.20 a	0.81 ab	49.00 b	1.18 b
		K2	59.80 a	0.89 a	39.80 a	0.86 a	61.40 a	1.21 ab
		K3	58.20 a	0.92 a	37.60 ab	0.87 a	61.00 a	1.28 a

K0, K1, K2, and K3 indicated that K fertilizer rates were 0, 120, 240, and 360 kg ha^−1^, respectively. For each cultivar, values followed by different letters within the same column are significantly different among potassium treatments at the 0.05 probability level.

### 3.3 Effect of potassium application on the anatomical structure of potential storage roots during storage root formation

**Figure 1:**
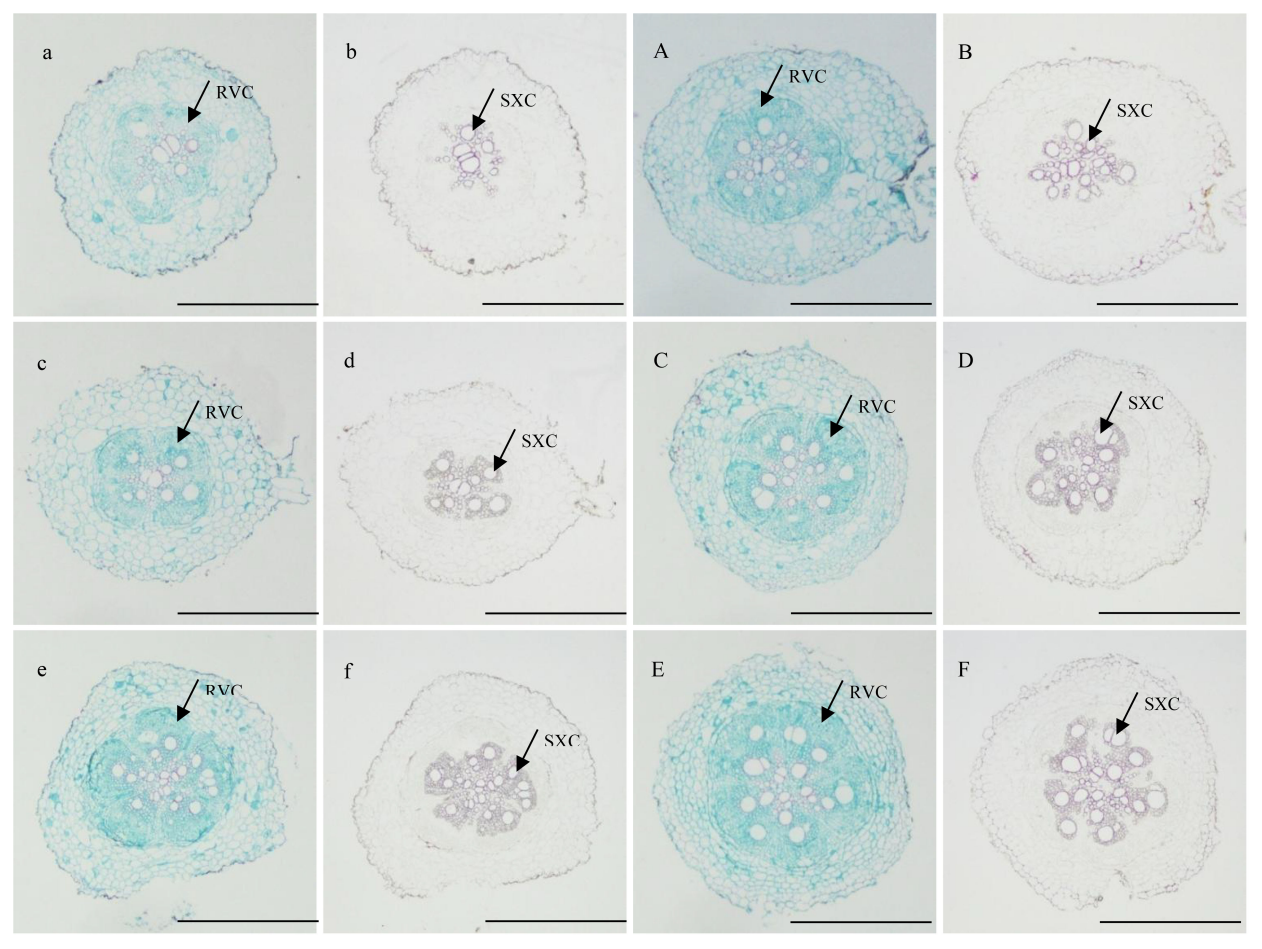
Cross-sectional view of sections of potential storage root stained with safranin-fast green and phloroglucin reagent of K2 at 10, 15, and 20 DAP (BJ553). (**A**, a, **C**, c, **E**, e) showed safranin-fast green stain of potential storage root sections at 10, 15, and 20 DAP, respectively; (**B**, b, **D**, d, **F**, f), and f showed phloroglucinol stain of potential storage root sections at 10, 15, and 20 DAP, respectively. SXC, secondary xylem conduits; RVC, complete regular vascular cambium. Scale bar = 500 μm.

At 10 DAP, the primary formation layer was more active at K2 than at K0, as K0 formed a semicircle around the phloem, while K2 formed almost a circle (**Figures 1a, A**). Meanwhile, the number of primary cambium cell layers and secondary xylem conduits of K2 increased (**Figure 1**). At 15 DAP, the primary form a ring, and K2 treatment significantly increased stele diameter, number of primary cambium cell layers, and secondary xylem conduit number (**Figures 1c, C** and **Table 6**). At 20 DAP, the diameter of the root, the secondary xylem conduit number, the diameter of the middle column, and the proportion of the middle column to the cross-section of the root exhibit a continuous increase, with significantly higher ratios observed in K2 treatment compared to K0 treatment, and the starch grains appeared in the parenchymal cells outside the primary of K2 (**Figures 1e, E** and **Table 6**).

**Table 6:** Quantitative indices of the internal structure of potential storage root internal structure at 10, 15, and 20 days after planting (BJ553).

Days after planting (days)	Treatment	RD (mm)	SD (mm)	PSCS (%)	NPL	PSN	SXCN
10	K0	0.94 b	0.48 b	26.27 a	3.00 b	5.00 a	4.00 b
	K2	1.08 a	0.55 a	26.13 a	3.67 a	5.00 a	12.00 a
15	K0	1.10 a	0.53 b	23.32 b	3.00 b	5.00 a	10.00 b
	K2	1.13 a	0.65 a	33.47 a	4.00 a	5.00 a	14.00 a
20	K0	1.18 a	0.73 b	38.19 b	4.00 b	5.00 a	20.00 b
	K2	1.29 a	0.85 a	43.13 a	5.00 a	5.00 a	24.00 a

K0, K1, K2, and K3 indicated that K fertilizer rates were 0, 120, 240, and 360 kg ha^−1^, respectively. Values followed by different letters within the same column are significantly different among potassium treatments at the 0.05 probability level.

RD, root diameter; SD, stele diameter; PSCS, the percentage of the stele in the cross-section; NPL, number of primary formation layers; NP, number of protoxylem; SXCN, secondary xylem conduits number; PSN, primary xylem number.

### 3.4 Effect of potassium application on lignin metabolism of potential storage roots during storage root formation

#### 3.4.1 Lignin content and its components

**Figure 2:**
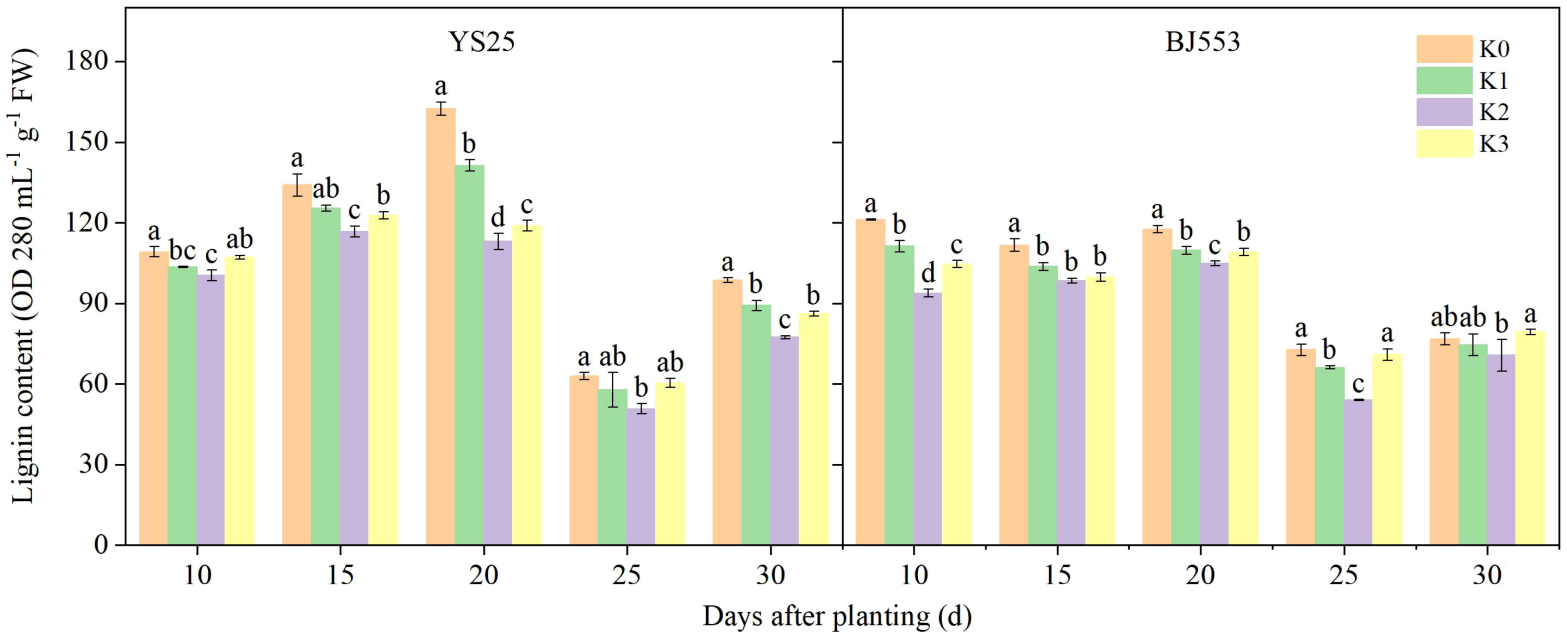
Lignin content in potential storage roots (2018). Error bars represent standard deviation (*n* = 3). Different lowercase letters indicate a significant difference among K treatments (*p* < 0.05).

The lignin content in the potential SRs peaked at 10–20 DAP, reached its lowest point at 25 DAP, and then increased (**Figure 2**). Lignin is generally accepted to form by polymerization of lignin monomers (Lee et al., 2021). The application of potassium resulted in a reduction in lignin content, with the most significant effect observed in the K2 treatment compared to the control treatment (except BJ553 at 30 DAP). The decrease in YS25 was greater than that of BJ553 on average. However, at 10 DAP, there was a significant decrease in lignin content for BJ553 of K2 treatment, whereas for YS25, this decrease was delayed until 20 DAP.

**Figure 3:**
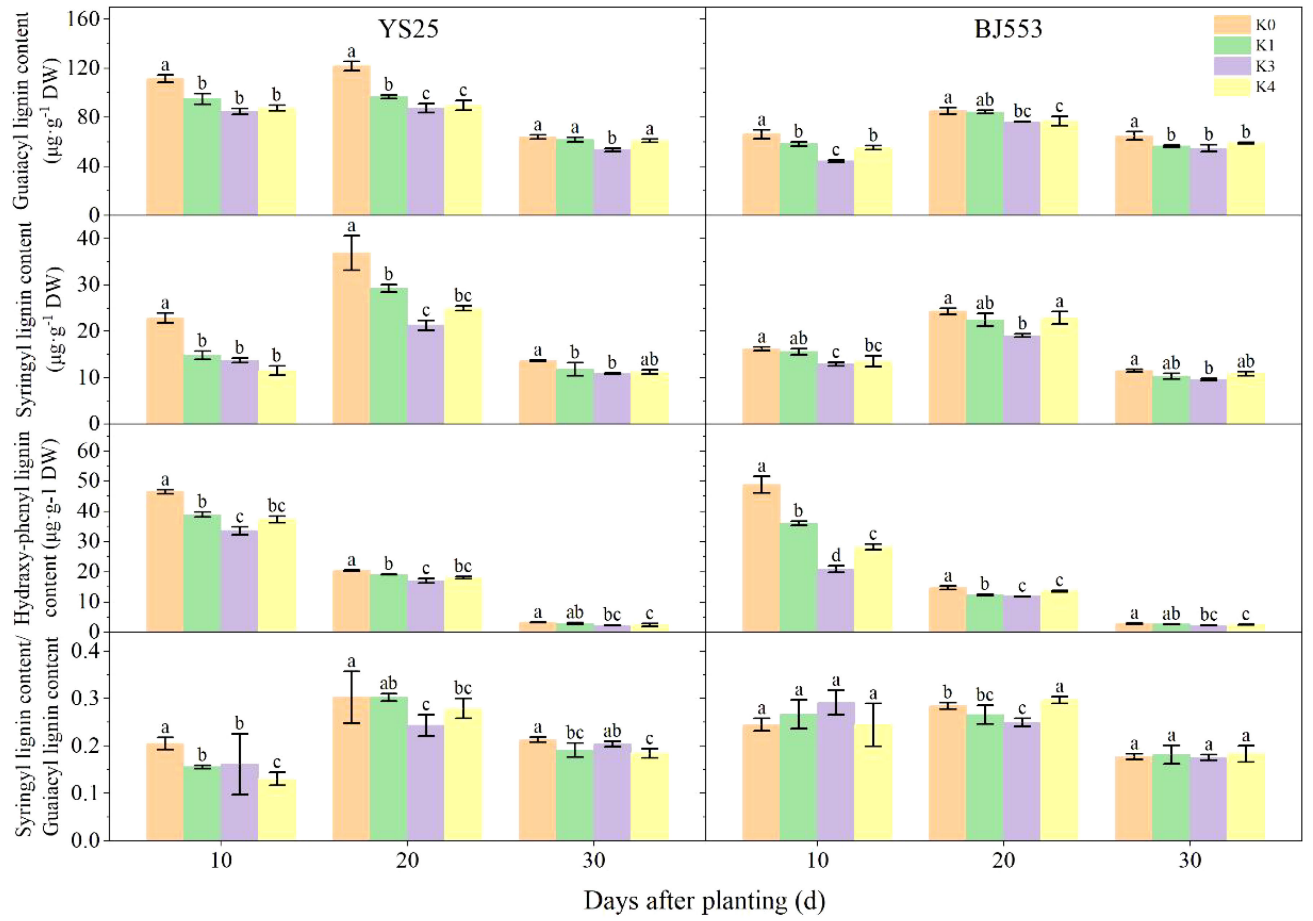
Lignin monomers content in potential storage roots (2018). Error bars represent standard deviation (*n* = 3). Different lowercase letters indicate a significant difference among K treatments (*p* < 0.05).

The changes in G-type and S-type lignin contents were consistent with the overall lignin content, while the H-type lignin content decreased as the potential SRs growth (**Figure 3**). Among the three monomers, G-type lignin appeared the highest content. The application of potassium led to a decrease in monomer contents, with significant changes observed in the K2 treatment. The great reduction of three monomers appeared at 10 DAP of BJ553, whereas the great reduction of G-type and S-type appeared at 20 DAP and that of H-type lignin content appeared at 30 DAP of YS25. The S/G ratio decreased after potassium application, with K2 showing the greatest reduction; YS25 and BJ553 exhibited reductions of 22.58% and 10.71%, respectively, at 20 DAP.

#### 3.4.2 Lignin biosynthesis-related enzyme activities

**Figure 4:**
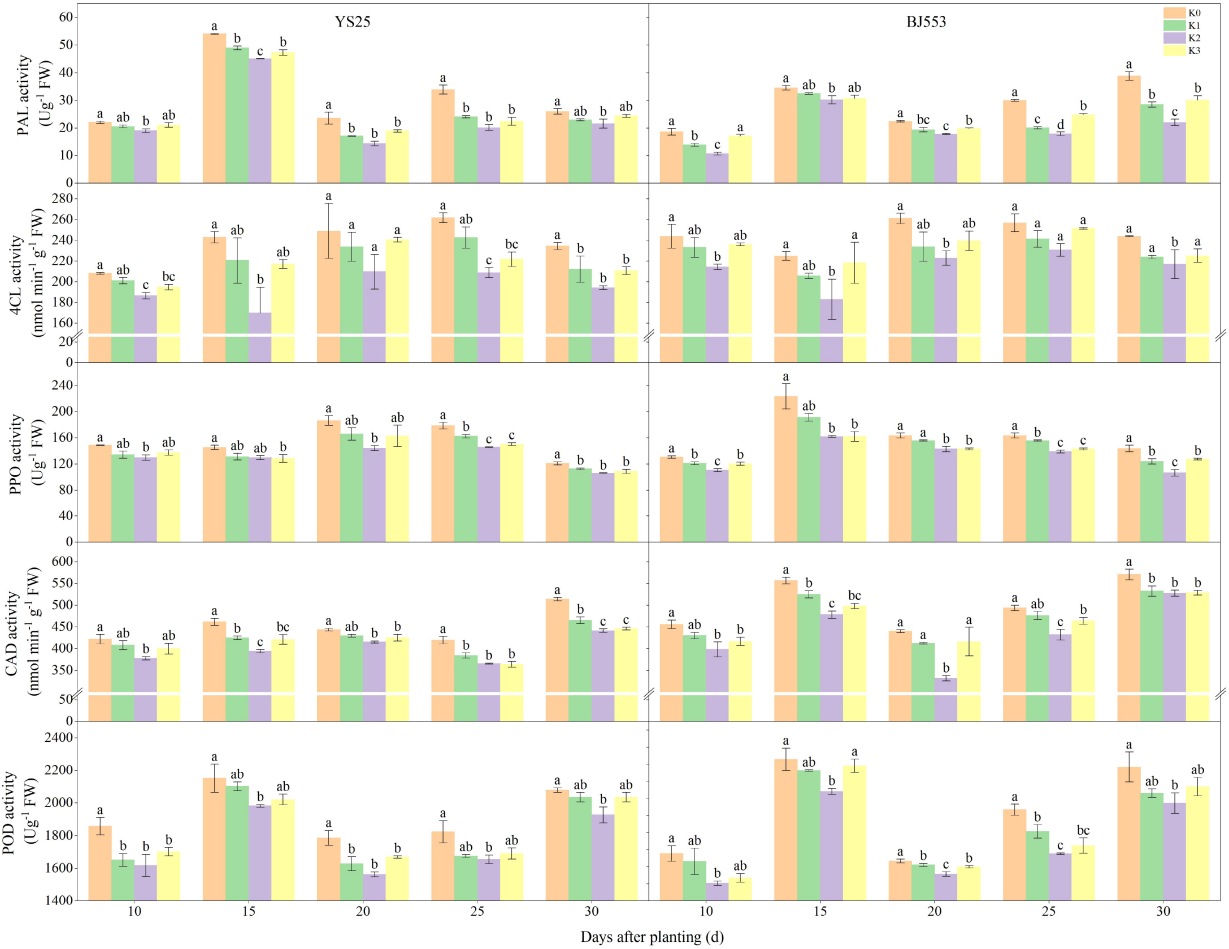
Lignin biosynthesis-related enzyme activity in potential storage roots during storage root formation (2018). Error bars represent standard deviation (*n* = 3). Different lowercase letters indicate a significant difference among K treatments (*p* < 0.05).

The PAL activity of YS25 at 15 DAP is significantly higher compared to that of BJ553, whereas the PPO, CAD, and POD activities are considerably lower in YS25 than in BJ553. During SR formation, the potassium application reduced the activities of PAL, 4-coumaric 4-CL, PPO, and POD in the potential SRs, and K2 treatment showed the greatest reduction, reaching a significant level compared to the K0 treatment (**Figure 4**). The PAL activity of BJ553 exhibited a significant increase at 10 DAP, whereas that of YS25 showed a delayed response until 20 DAP.

## 4 Discussion

### 4.1 Effect of potassium application on the number of storage roots and its relationship with storage root appearance quality

The price of fresh sweet potatoes is determined by their appearance quality (Su and Sun, 2019; Hou et al., 2020), which is assessed based on the size, shape, and smoothness of their skin, with a greater emphasis on size and uniformity. The acceptance of SRs by consumers is generally higher when they are medium-sized (100–500 g), are spindle-shaped, and have a bright skin. Conversely, the merchantability of SRs is not improved when they are oversized or undersized (Villordon et al., 2009; Su and Xue, 2021). The SR number plays a pivotal role in determining both the yield and appearance quality (Si et al., 2022), and potassium application could regulate the SR number by affecting sucrose metabolism (Du et al., 2020). However, the impact of potassium fertilizer on the homogeneity of SRs has received limited attention in previous studies. The present study demonstrated that K2 treatment significantly increased SR yield and commercial SR yield at harvest time (**Table 2**), which are consistent with previous findings (Villordon et al., 2009; Su and Xue, 2021). The number of SRs per plant and the weight of single SRs also exhibited an increase. Notably, the increment in the number of SRs was significantly greater than that in single SR weight during 2017 (**Table 2**). The highest medium SR yield (**Table 3**) and the lowest coefficient of variation of SR weight, length, and width appeared in K2 treatment as well (**Table 4**). The coefficient of variation of SR width had significant negative correlation with both SR no. and yield (**Supplementary Table 1**). A previous study proved that proper phosphorus application improved the SR appearance quality by increasing the length/diameter ratio and uniformity of SR weight (Si et al., 2022). In conclusion, the application of potassium fertilizer significantly increased single SR yield and the number of SRs per plant, promoting size uniformity and improving the weight and proportion of medium-sized SRs. Additionally, it enhanced root elongation and optimized SR shape, collectively improving the appearance quality of SRs.

### 4.2 Effect of potassium application on lignin metabolism in potential storage roots and its relationship with storage root formation

The anatomical structure of roots is crucial for SR formation. The roots with strong primary cambium meristematic ability and weak columnar sheath cell lignification were more likely to differentiate into SRs (Tanaka et al., 2005; Villordon et al., 2009; Tanaka, 2016). The phloroglucinol staining results revealed a significant increase in both the number and distribution area of secondary xylem conduits under K2 treatment at 10, 15, and 20 DAP (**Table 6** and **Figures 1B, b, D, d, F, f**), which can selectively stain the lignin of cell. The lignin content in the potential SRs was lower in K2 treatment than in K0 treatment (**Figure 2**). That is, the lignification level of column cells in potential RTs was reduced in K2 treatment. The increase in stele diameter and the proportion of the stele in the cross-section proved the above conclusion (**Table 6**). Interestingly, the potential SR diameter of K2 was greater than that of K0 treatment at the same time (**Table 5**). Previous studies have proved that root lignification increased with lignin accumulation (Jia et al., 2015), while downregulation of lignin content at the early stage of SR formation facilitates the differentiation of ARs into SRs (Firon et al., 2013; Kim et al., 2015). The G-type and S-type lignin contents and S/G ratio in transgenic sweet potato plants overexpressing IbCAD1 were higher than that in non-transgenic plants, but root fresh weight and storage roots formation rates were lower (Lee et al., 2021). The results of our study revealed a significant positive correlation between the monomer contents and the lignin content (**Supplementary Table 1**). The application of moderate potassium significantly reduced the lignin content, as well as the contents of all three monomers and the S/G ratio during storage root formation in potential SRs (**Figures 2**, **3**). Among the three monomers, G-type lignin content exhibited the highest reduction, with both G-type and H-type lignin contents showing greater average reductions compared to H-type lignin content (**Figure 3**). A previous study has demonstrated that lignification initiates with the precipitation of G-type lignin accumulation (Berthet et al., 2011; Schuetz et al., 2014). Consequently, the application of moderate potassium resulted in a decrease in lignin and monomer accumulation, leading to a reduction in the degree of stele cell lignification and an improvement in the rate of SRs formation.

PAL, 4-CL, CAD, PPO, and POD have been reported to play pivotal roles in the biosynthesis of lignin (Jia et al., 2015; Liu et al., 2018; Vanholme et al., 2019; Zhang et al., 2022). The potential SRs exhibited low levels of POD and PPO activities, as well as the lowest concentrations of lignin and phenolics. However, they displayed high levels of pedunculated roots and fibrous roots (Kim et al., 2015). PAL, 4-CL, and CAD genes were downregulated in potential SRs during SRs formation as well (Firon et al., 2013; Tanaka, 2016). We found that the activities of CAD and PPO exhibited a highly significant positive correlation with lignin content. Moreover, the activities of 4-CL, CAD, and PPO showed an extremely significant positive correlation with G-type and H-type lignin contents. Additionally, CAD activity demonstrated a remarkably significant negative association with the number of SRs per plant (**Supplementary Table 1**). After potassium fertilization, the activities of PAL, 4-CL, PPO, CAD, and POD exhibited a decrease. Notably, the reduction in activity was significantly more pronounced in the K2 treatment compared to the K0 treatment (**Figure 4**). Upon comparison of the two variants, a significant increase caused by K2 treatment in PAL activity was observed in BJ553 at 10 DAP, which coincided with the initial stages of SR formation, accompanied by consistent trends in lignin and G-type contents. Conversely, YS25 exhibited a substantial increase at 20 DAP, which coincided with the late stages of SR formation, accompanied by consistent trends in lignin and G-type contents (**Figures 2**–**4**). The greater change range of the SR number of BJ553 compared to YS25 after the application of potassium fertilizer may be attributed to this factor. In essence, the application of potassium fertilizer primarily promoted adventitious root differentiation by inhibiting lignin biosynthesis in potential SRs. The timing of potassium-regulated lignin metabolism in BJ553 roots occurred earlier, leading to a significantly greater augmentation in SR number compared to YS25.

## 5 Conclusion

Regulating the SR numbers of sweet potato is crucial for improving both yield and appearance quality synergistically. Potassium fertilization significantly increased SR numbers, leading to an increase of commercial and medium-sized SRs yield, with the most notable effect observed in K2 treatment. Proper potassium application resulted in a decrease in the activity levels of enzymes involved in lignin biosynthesis, inhibiting the accumulation of lignin and its monomers. This led to reduced lignification within potential storage roots’ stele and promoted an increase in the ratio of mid-column diameter area to total root diameter area, facilitating storage root formation. The effect of potassium on lignin metabolism in BJ553 roots was earlier and resulted in a greater increase in the SR number compared to YS25.

## Data availability statement

The original contributions presented in the study are included in the article/**Supplementary Material**. Further inquiries can be directed to the corresponding author.

## Author contributions

B-KL: Writing – original draft, Data curation, Investigation, Software. B-JX: Writing – review & editing. C-CS: Writing – review & editing. W-QS: Investigation, Writing – original draft. G-ZD: Investigation, Data curation, Writing – original draft. L-XT: Writing – original draft. XM: Methodology, Writing – original draft. C-YS: Supervision, Funding acquisition, Writing – review & editing. H-JL: Writing – review & editing, Funding acquisition.
