# Supplementary material for: Effect of potassium fertilization on storage root number, yield, and appearance quality of sweet potato (Ipomoea batatas L.)
Source: Front Plant Sci. 2024 Feb 22;14:1298739. doi: 10.3389/fpls.2023.1298739 (PMC10917953; doi:10.3389/fpls.2023.1298739)
Supplement: Supplementary file 1 [file DataSheet_1.pdf]

# *Supplementary Material*

## 1 SupplementaryTable

**SupplementaryTable 1** Correlation analysis between the number of single plants bearing storage roots and the main agronomic traits and lignin and its related enzymes (YS25 and BJ553)

| Items | Yield | SRs NO. | Potential SRs diameter | Potential SRs NO. | Lignin | G | S | H | PAL | 4-CL | CAD | POD | PPO | S/G | SRs length CV | SRs diameter CV |
|---|---|---|---|---|---|---|---|---|---|---|---|---|---|---|---|---|
| Yield | | .980* | .980* | .999** | -.966* | -.985* | -.985* | -.987* | -0.895 | -.982* | -.981* | -.952* | -.983* | -.979* | -0.702 | -.952* |
| SRs NO. | .980* | | 0.921 | .983* | -.996** | -.981* | -.970* | -.988* | -0.942 | -.969* | -.996** | -.982* | -.987* | -0.923 | -0.812 | -.966* |
| Potential SRs diameter | .980* | 0.921 | | .974* | -0.899 | -.952* | -.965* | -0.948 | -0.819 | -.961* | -0.929 | -0.888 | -0.944 | -.998** | -0.572 | -0.907 |
| Potential SRs NO. | .999** | .983* | .974* | | -.967* | -.979* | -.977* | -.982* | -0.887 | -.974* | -.980* | -0.948 | -.979* | -.971* | -0.698 | -0.944 |
| Lignin | -.966* | -.996** | -0.899 | -.967* | | .982* | .969* | .988* | .967* | .969* | .997** | .994** | .989* | 0.905 | 0.857 | .978* |
| G | -.985* | -.981* | -.952* | -.979* | .982* | | .998** | .999** | .955* | .998** | .993** | .985* | .999** | .962* | 0.795 | .991** |
| S | -.985* | -.970* | -.965* | -.977* | .969* | .998** | | .995** | 0.941 | 1.000** | .985* | .974* | .995** | .975* | 0.764 | .986* |
| H | -.987* | -.988* | -0.948 | -.982* | .988* | .999** | .995** | | .955* | .995** | .997** | .988* | 1.000** | .956* | 0.802 | .989* |
| PAL | -0.895 | -0.942 | -0.819 | -0.887 | .967* | .955* | 0.941 | .955* | | 0.945 | .961* | .987* | .960* | 0.841 | 0.936 | .984* |
| 4-CL | -.982* | -.969* | -.961* | -.974* | .969* | .998** | 1.000** | .995** | 0.945 | | .984* | .976* | .995** | .972* | 0.771 | .988* |
| CAD | -.981* | -.996** | -0.929 | -.980* | .997** | .993** | .985* | .997** | .961* | .984* | | .992** | .997** | 0.935 | 0.826 | .985* |
| POD | -.952* | -.982* | -0.888 | -0.948 | .994** | .985* | .974* | .988* | .987* | .976* | .992** | | .990** | 0.901 | 0.884 | .993** |
| PPO | -.983* | -.987* | -0.944 | -.979* | .989* | .999** | .995** | 1.00** | .960* | .995** | .997** | .990** | | .953* | 0.811 | .991** |
| S/G | -.979* | -0.923 | -.998** | -.971* | 0.905 | .962* | .975* | .956* | 0.841 | .972* | 0.935 | 0.901 | .953* | | 0.6 | 0.923 |
| SRs length CV | -0.702 | -0.812 | -0.572 | -0.698 | 0.857 | 0.795 | 0.764 | 0.802 | 0.936 | 0.771 | 0.826 | 0.884 | 0.811 | 0.6 | | 0.859 |
| SRs diameter CV | -.952* | -.966* | -0.907 | -0.944 | .978* | .991** | .986* | .989* | .984* | .988* | .985* | .993** | .991** | 0.923 | 0.859 | |

Note: * and ** indicate significant differences at 0.05 and 0.01 probability levels, respectively.

## 2 Supplementary Figures

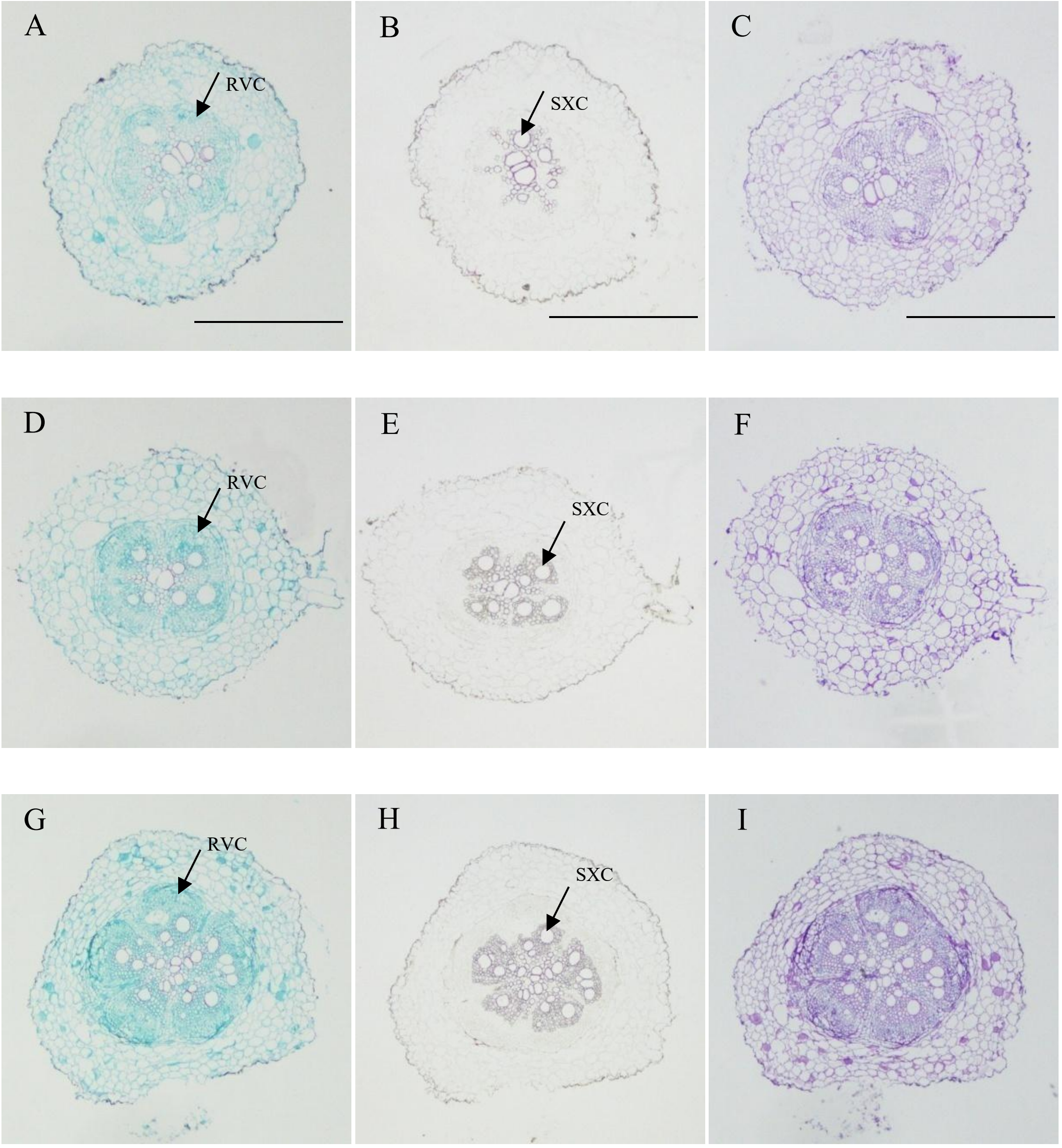


Supplementary Fig.1 Cross-sectional view of sections of potential storage root stained with Safranine O-Fast Green, resorcinol, and Schiff's periodate of K0 at 10、15、20 DAP (BJ553)

A, D, and G showed Safranine O-Fast Green stain of potential storage root sections at 10, 15, and 20 DAP, respectively; B, E, and H showed phloroglucinol stain of potential storage root sections at 10, 15, and 20 DAP, respectively; C, F, and I showed Schiff's periodate stain of potential storage root sections at 10, 15, and 20 DAP, respectively.

SXC, secondary xylem ducts; RVC, complete regular vascular cambium. Scale bar = 500μm
